# Out-of-Plane Flexural Behavior of Insulated Wall Panels Constructed with Large Insulation Thicknesses

**DOI:** 10.3390/ma16114160

**Published:** 2023-06-02

**Authors:** Jacob Luebke, Fray F. Pozo-Lora, Salam Al-Rubaye, Marc Maguire

**Affiliations:** 1 Structural Design Department, Calder Richards Structural Consulting Engineers, Salt Lake City, UT 84104, USA; 2 Durham School of Architectural Engineering & Construction, College of Engineering, University of Nebraska-Lincoln, Omaha, NE 68182, USA; salrubaye@unomaha.edu

**Keywords:** thermal efficiency, sustainability, fiber-reinforced plastic (FRP) shear connectors, sandwich wall panels

## Abstract

Insulated concrete sandwich wall panels (ICSWPs) are gaining popularity as energy regulations become stricter worldwide. ICSWPs are now being constructed with thinner wythes and thicker insulation to keep up with the changing market, which is reducing material costs and increasing thermal and structural efficiency. However, there is a need for adequate experimental testing to validate the current design methods for these new panels. This research aims to provide that validation by comparing the predictions of four different methods with experimental data obtained from six large-scale panels. The study found that while current design methods adequately predict the behavior of thin wythe and thick insulation ICSWPs within the elastic region, they do not accurately predict their ultimate capacity.

## 1. Introduction

Insulated concrete sandwich wall panels (ICSWPs) are becoming more popular as the industry continues to move toward more thermally efficient buildings [1,2]. ICSWPs consist of two wythes of concrete that sandwich a layer of insulation. The sandwiched layer of insulation provides an increased thermal efficiency sought after in thermally efficient buildings. A shear connector is a hardware that bridges the two wythes and transfers forces through its connection. ICSWPs can be designed as non-composite, fully composite, or partially composite. These categories indicate the degree to which the two concrete wythes act in unison to resist loads. However, the extremes of composite behavior, non-composite and fully composite, suppose an unrealistic performance of the ties. Non-composite implies no force transfer or stiffness by the connector, whereas the fully composite behavior involves an infinitely rigid tie [3]. This is the main reason why ICSWPs with FRP connectors are designed to behave as partially composite. Designing in such a manner allows engineers to optimize the structural capacity of the panels and reduce both construction costs and energy consumption during building operations.

As partially composite ICSWPs become more popular and designs push the boundaries, more effort has been made to develop methods to design and analyze these structural panels. Many of the current and past analysis and design methods have been verified by comparing their results to existing ICSWP testing in the literature [4,5,6,7,8,9,10]. One of the earliest attempts to describe the ICSWP behavior can be attributed to Holmberg and Plem [4] and Allen [5]. The former study was exclusively devoted to the behavior of sandwich panels made of concrete with steel truss connectors, whereas the latter was a generalized review of the sandwich behavior for panels made of several configurations. Reference [11] verified both methodologies for the elastic range, generalized the method in [4], and determined they were appropriate for use in partially composite ICSWP elastic design. However, due to the complex nature of the sandwich panel behavior, the analysis methods, and the difficulties of incorporating them in the design process, either a simplified or finite element analysis approach is currently used in the industry [12]. Typical methods employed in the industry for designing these panels include the Percent Composite Approach (PCA), the Sheaf Flow Approach, and some variations on sandwich beam theory [3,8,9,13,14,15,16].

Aside from being structurally efficient, insulated concrete sandwich wall panels are also thermally efficient. The original purpose of ICSWP members was to provide integrated insulation in the structural member without affecting the structural performance [17]. Early-generation sandwich wall panels comprised particular types of connectors, such as steel bars that penetrated the insulation and steel trusses placed at foam terminations in the panel. Solid sections interrupting the insulation were often employed, disregarding the thermal bridge they created [18]. Solid sections of concrete are still common for structural reasons, but thermally conscious engineers and architects are making an effort to reduce them [18]. Many solutions have been developed to increase the thermal efficiency of ICSWP, including changing the tie material, reducing other bridges, and rethinking the general configuration [19,20,21,22,23,24,25].

Current-generation panels incorporate ties made of FRP composites, which reduce thermal bridging between the two concrete wythes [22,23,26,27,28]. However, this behavior is only warranted if both the insulation and the tie have the same thermal conductivity. These newer, often proprietary, FRP ties also provide various degrees of composite action depending on the number of ties employed and their stiffness. Commercially, there are few wythe connectors available for large insulate thicknesses, up to a maximum insulation of 125 mm. Although the literature contains FRP connector prototypes developed for bridging up to 300 mm of insulation and others that would require minimum modification, such hardware lacks large-scale testing or real case studies using such large connectors [29,30]. Another relevant issue regarding current-generation panels is thermal bowing, which is expected due to the heat transfer elimination between two concrete wythes. Although many attempts have been made to test panels for thermal loading, to account for this type of problem in design, and to predict behavior, there is no consensus in the literature or US building code on the appropriate way to account for it in design [4,31,32]. Notably, the only common ground for the literature on thermal bowing is that panels will crack, and panel-to-panel connections may become damaged if thermal gradients are not considered early in the design process.

As code regulations become stricter regarding energy loss through the building envelope, thermal efficiency has become a concern to building owners, architects, and the energy code. Moreover, as energy production remains steady and the population increases, enhancing thermal efficiency has become a top priority for building owners and governments trying to cut energy spending and seek to foster new energy sources. Table 1 shows the variation in R-value for a given insulation thickness. As this table shows, thermal efficiency will roughly increase by 25% for every 50 mm increase in insulation thickness if one could only develop a practical shear tie to connect the two concrete wythes.

**Figure 1:**
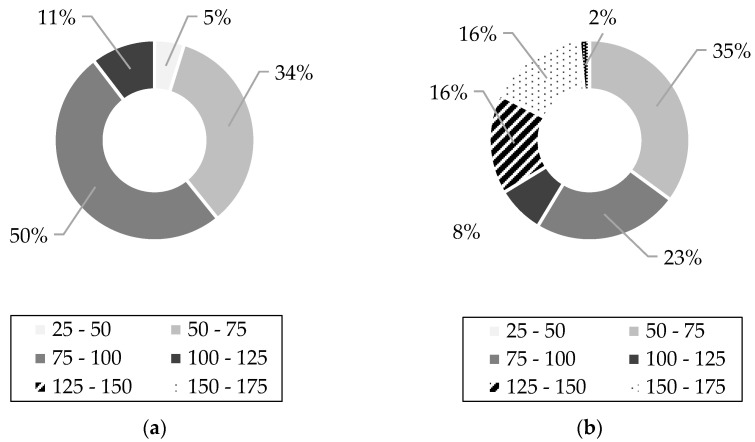
Pie charts summarizing the available testing data in the literature regarding push-through testing (**a**) and large-scale flexural testing (**b**). Each slice division represents the thickness range in mm.

Moreover, structural efficiency will also increase if the insulation thickness increases and the concrete wythes retain their properties due to an increase in the distance between the steel gravity center and the neutral axis of the section. However, such an increase necessitates adequate shear tie stiffness, which is currently scarce in both real practice and research. In fact, much of the testing data consists primarily of panels and push-through specimens with insulation thicknesses between 50 and 100 mm. Among the available testing data in the published literature are 365 double shear or push-through specimens and 137 panels [7,13,16,22,24,27,29,30,33,34,35,36,37,38,39,40,41,42,43,44]. A pie chart summarizing this list, which is not exhaustive, is provided in Figure 1. This list was stratified by wythe thickness and insulation thickness to better understand the scope of the existing testing data available in the literature regarding wythe and insulation thickness. Only 16% of the panels in the literature have insulation thicknesses that do not fall within two to four inches. This range of insulation thickness is still the most prevalent for push-through specimens as well, accounting for 58% of all push-through specimens tested. Furthermore, there are a limited number of commercial connectors available that can be used for insulation thicknesses greater than 200 mm.

The percentages represent how much of the testing in the literature has been devoted to that particular concrete thickness range in mm.

With the above in mind, this study seeks to better understand the behavior of insulated concrete sandwich panels in flexure constructed using large insulation thicknesses and connectors for the purpose of design and field implementations. More specifically, this study evaluates the following:

1. The impact of increasing insulation thickness on the double shear test performance of the tie, global flexural behavior of ICSWPs, and local behavior at the vicinity of connectors.
2. Comparison of the results of this research to results in the literature and analytical methods for computing tie performance alone and global flexural behavior of large-scale specimens.
3. Evaluation of the impact of increasing the insulation thickness on the “percent composite action” of large-scale specimens to assess whether the connector implemented in the study can effectively maintain significant composite action for the panels.

## 2. Materials and Methods

The experimental program of this study comprised testing twelve double shear specimens and six large-scale partially composite concrete sandwich wall panels. These tests were conducted to analyze the viability of and to verify the current methods of design for partially composite ICSWPs whose wythes and insulation thicknesses lie on the extreme ends of the currently available testing data in the literature. These extreme ends include concrete wythe thicknesses of two inches and insulation thicknesses of eight and ten inches. The primary concern was the ability of double shear test data to be used in available elastic methods and strength methods that are common to US practice for the extreme wythe thicknesses identified. This section depicts the materials used, the testing layout, sensors, and procedures implemented for testing these twenty-one specimens, as well as providing the specimen fabrication information and configurations.

### 2.1. Double Shear Test: Materials, Specimens, Setup, and Instrumentation

**Figure 2:**
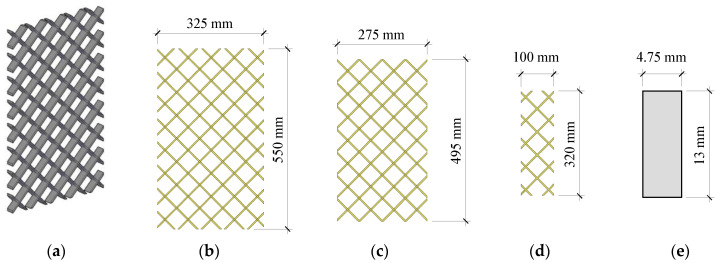
GFRP Ties: 3-D view of the grid (**a**), connector implemented with 250 mm insulation (F250) (**b**), connector implemented with 200 mm insulation (F200) (**c**), connector implemented with 50 mm insulation (F50) (**d**), enlarged cross-section of the ribs (**e**).

**Figure 3:**
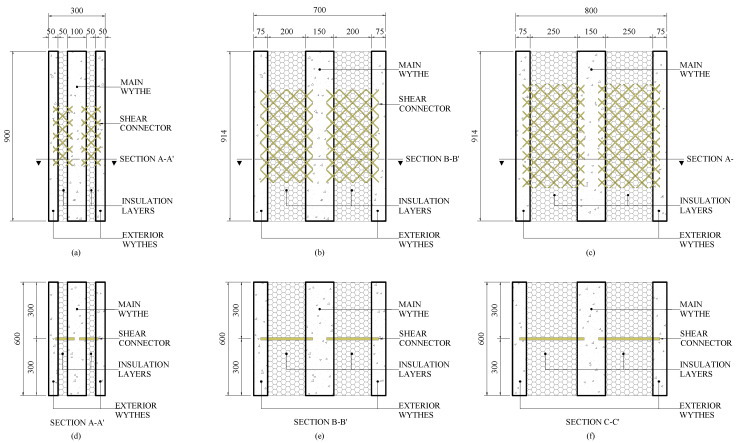
Double shear test specimens for connectors F50-F250 (**a**–**c**) and their corresponding sections (**d**–**f**). Units in mm.

Three different connector configurations were used for the double shear and large-scale test specimens. Non-commercial connectors were created because there were no commercial connectors available in the US for the panel geometries under investigation. These connectors were classified and designed based on the thickness of the insulation that they bridged. Custom connectors were created for the varying insulation thicknesses since commercially available connectors were unable to bridge the large insulation thicknesses desired. These connectors were all made of a 1.2 m × 3.60 m GFRP grate that contained rectangular bars with a cross-sectional dimension of 4.75 × 13 mm² spaced 50 mm on center (OC) in both perpendicular directions. The grate was shaped into three connectors that were designated as F250, F200, and F50 for insulation thicknesses of 250, 200, and 50 mm, respectively, see Figure 2. Embedment depths of ties into concrete were selected as 37.5 mm on each end for the F250 and F200 connectors and 25 mm for the F50 connector. The design and construction of the double shear specimens are like many of those described in the literature [22,33,34]. The double shear specimens all measured two feet in width and three feet in height. The thicknesses of each specimen and their wythes are listed in Figure 3. A single lifting anchor was placed in the center wythe on the top of the specimens and was used to move the specimen before and after testing (not pictured).

**Figure 4:**
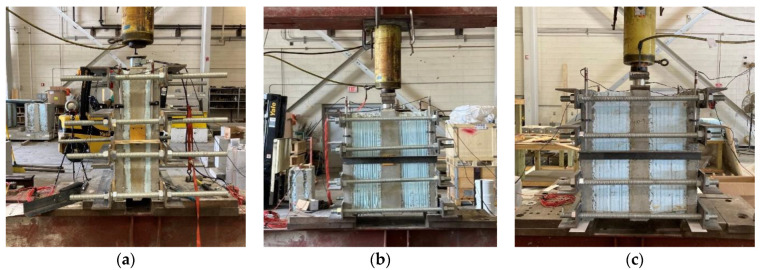
Test setup of double shear specimens: F50 (**a**), F200 (**b**), F250 (**c**).

Testing the specimens consisted of supporting them along their length on both outer wythes with two 2″ thick steel plates leaving the inner wythe free to displace under axial load. Two polytetrafluoroethylene (PTFE) strips were placed between the steel plates and the outer wythes to reduce friction. The specimens were loaded using a 150-ton hydraulic ram (Enerpac, Menomonee Falls, WI, USA), and a load cell sandwiched (BDI Systems Ltd., Louisville, CO, USA) between steel plates centered on the inner wythe was used to record the load. Relative displacement was measured using four separate Linear Variable Differential Transformers (LVDTs) (BDI Systems Ltd, Louisville, CO, USA). The measurements from these four LVDTs were averaged to find the relative displacement between the inner and outer withes, also known as slip in the literature. The LVDTs were attached to the outer wythes and were supported by either a steel angle or wood angle attached at the mid-height of the center wythe of the specimen. This angle was offset from the wythes to allow free movement as the center wythe displaced. All specimens were loaded until the ultimate strength of the shear tie was reached. Following the failure, each double shear was removed from the frame, and the insulation was removed to visually inspect the failure of the connectors. The placements of the load cell, plates, and LVDTs are shown in Figure 4. 

Braces to prevent out-of-plane movement of the outer wythes were installed at four equally spaced points along the height of the specimen. Each brace consisted of an L127 × 127 × 9.5 angle and two 25 mm diameter all-thread rods connected using four 10 mm diameter post-installed drill-in concrete anchors into the outside faces. The purpose of these braces was to keep the wythes parallel and prevent “pinching” action known to reduce wythe connector strengths [28]. Keeping the wythes parallel is also a requirement of all historical analysis methods (e.g., [4,5,8,9,45,46]), so the pinching action should be limited.

### 2.2. Large-Scale Test: Materials, Specimens, Setup, and Instrumentation

Six large-scale panels measuring 6.7 m in length and 0.60 m in width were designed and constructed for testing the partially composite behavior of the panels both in strength and deflection. The intention of the program was to test panels of varying thickness with different levels of reinforcement. Due to material constraints, it was elected to maintain the same connector pattern in all specimens. 

**Figure 5:**
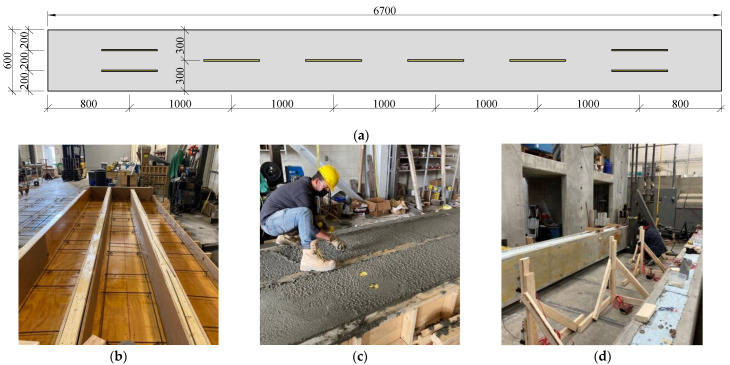
Planar view of the large-scale panel connector inserts locations (**a**), sandwich panels prior to the pour (**b**), lifting anchor placement at the end of concrete pour (**c**), and panel prior to testing (**d**). Units in mm.

Two panels were designed for an insulation thickness of 50 mm, two for an insulation thickness of 200 mm, and two for an insulation thickness of 250 mm. These thicknesses corresponded with the thicknesses of the insulation tested with the double shear specimens. The same connectors were utilized in the large-scale panels as used in their corresponding double shear specimens. Table 2 displays the various dimensions of the panels and their reinforcement in the longitudinal and transverse directions. Each panel contained eight connectors with two rows of connectors at the ends and one row of connectors throughout the center of the panel. The end connectors were placed 800 mm from the end of the panel to the center of the connectors. The inner connectors were all placed at 1.0 m center-to-center. Figure 5a shows where the cuts are located for each large-scale panel.

**Figure 6:**
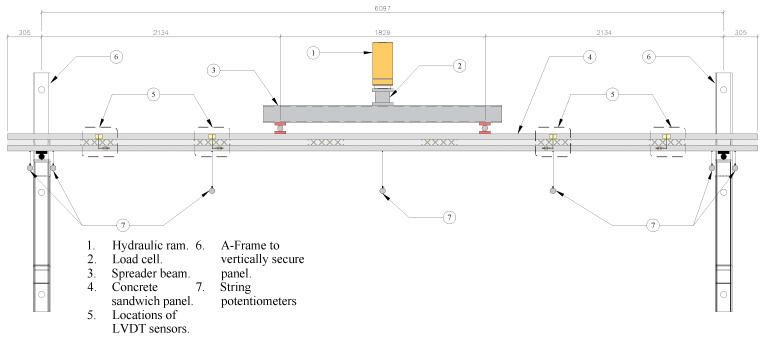
Plan view of the large-scale panel test setup. Units in mm.

Each large-scale panel was tested using two symmetric point loads, each located approximately 0.90 m from the center span. This loading provided a constant maximum moment throughout the center 1.80 m of the panel. A single ram was used to load the panel, and a Hollow Structural Section (HSS) spreader beam was used to split the load into the two-point loads with rollers at the contact points. A load cell sandwiched between steel plates was placed between the ram and spreader beam to measure the applied load. The number of plates varied to account for the differences in thickness between the two-inch insulation and the eight- and ten-inch insulation panels. The spreader beam, load cell, and plates all utilized polytetrafluoroethylene (PTFE) strips between them and their vertical supports to reduce friction during loading. This testing setup, depicting one of the two-inch thick insulation panels, is shown in Figure 6.

## 3. Results

Concrete cylinder testing was completed for both double shear specimens and the large-scale panels. The specimens were cast in three separate instances. The first instance consisted of seven double shear specimens. The second instance consisted of five double shear specimens and three large-scale panels, and the third instance consisted of the final three large-scale panels. Concrete cylinders were cast during each instance to determine the concrete material properties for all specimens. Cylinders were cast using concrete from the middle of each pour. Cylinders were tested for compressive stress for the double shear specimens. Compressive stress tests, modulus tests, and split tension tests were conducted on cylinders for large-scale panel specimens. The results of this testing are found in Table 3 and are used in the predictions of the following sections.

### 3.1. Double Shear Test Results

**Figure 7:**
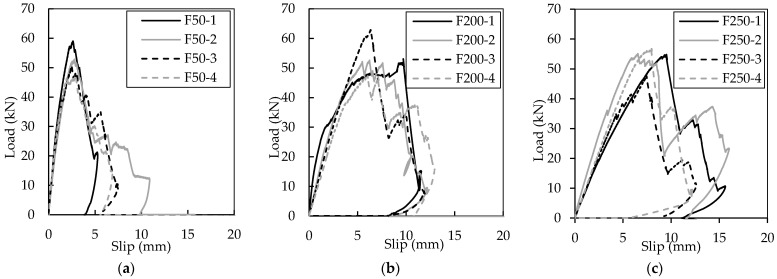
Double shear test results for specimens F50 (**a**), F200 (**b**), F250 (**c**).

**Figure 8:**
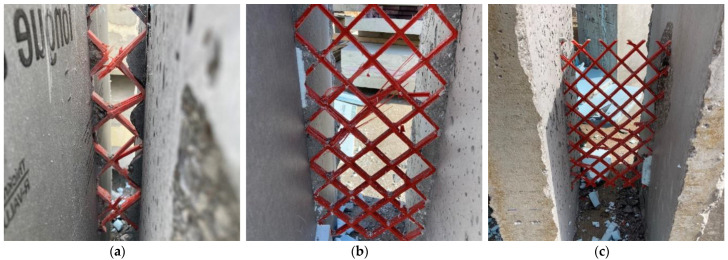
Failure modes of F50 (**a**), F200 (**b**), F250 (**c**).

Figure 7a–c shows the shear load and deflection relationship for all double shear specimens tested. All double shear specimens reached similar ultimate loads between 46 and 58 kN. The average maximum load for all specimens is shown in Table 4. For all samples, the specimen failures were controlled by connector failure. No concrete failure (i.e., break-out or punch-through) was observed in any specimen. In addition, in all but specimen F200-1 only the connector on one side of the specimen failed. Buckling of truss elements in compression was frequently observed, as can be seen in Figure 8a–c. Delamination of the GFRP truss elements in tension was also observed and is also shown in Figure 8b. GFRP delamination and possible buckling were observed to occur in the eight truss elements of each connector post mortem. Moreover, buckling was observed to be the more common of these two failure mechanisms. 

### 3.2. Large-Scale Test Results

**Figure 9:**
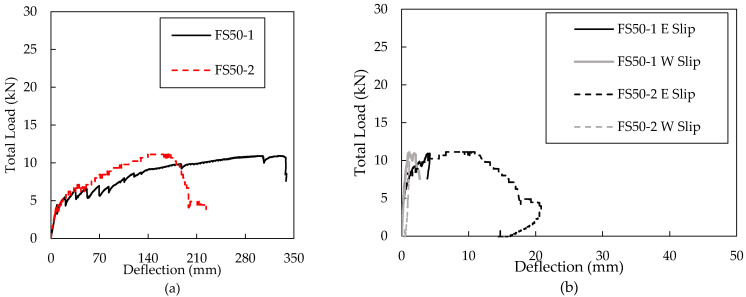

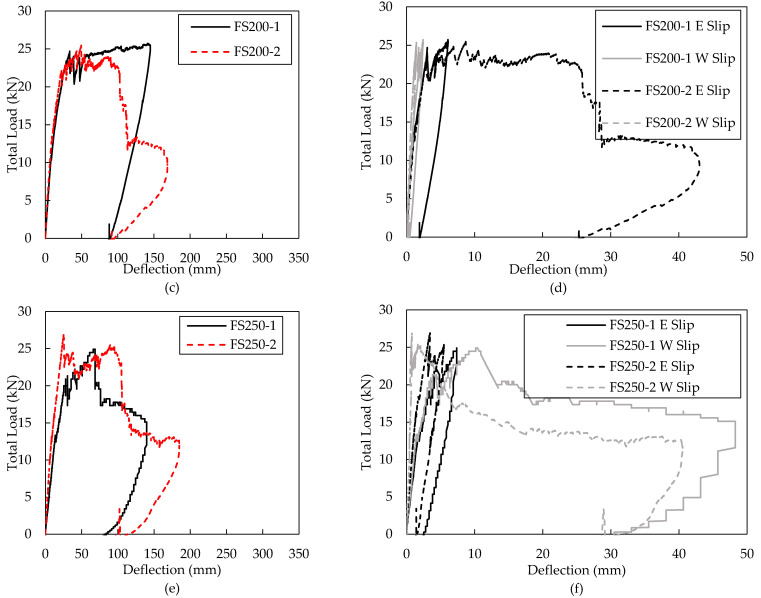
Load versus deflection and load versus slip plots for specimens FS50 (**a**,**b**), FS200 (**c**,**d**), FS250 (**e**,**f**).

Six large-scale panels were tested monotonically until failure. All panels of the same series achieved similar maximum loading by design; all maximum loadings are found in Table 5. The load and deflection relationship of each panel is plotted below in Figure 9. Deflection values were measured at multiple locations along the length and averaged from sensors placed at the top and bottom of the wythe. Deflections were measured at the supports to account for the settling of the panel into the testing mechanism and any support deflections of the A-frame support. The midspan deflections presented herein account for this settling, whereby the average midspan deflection was arithmetically subtracted from the average support settlement.

**Figure 10:**
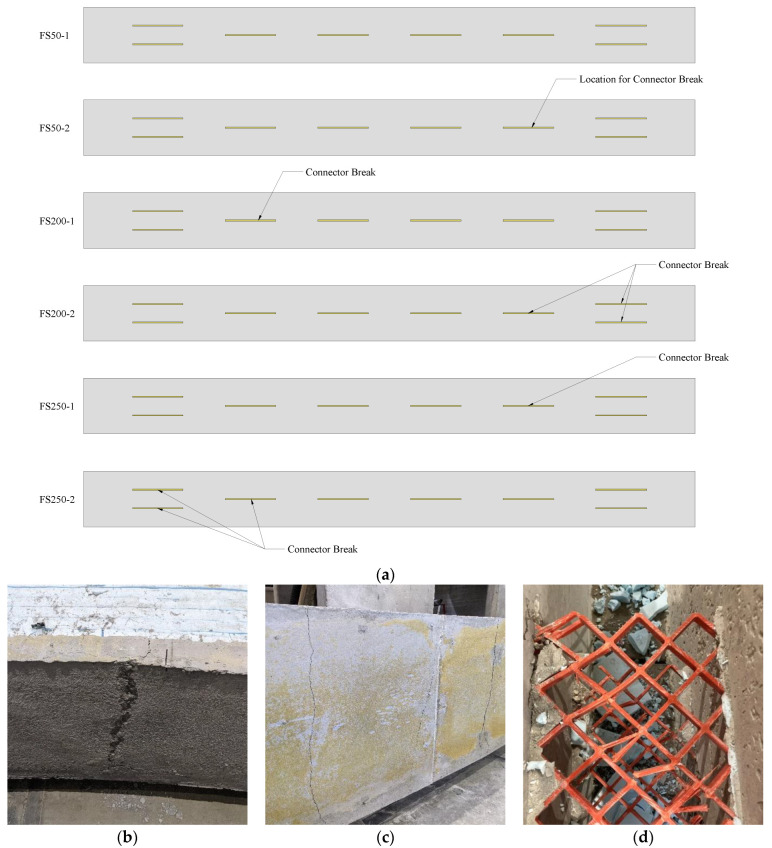
Location of observed connector failures (**a**), and examples of large-scale panel concrete crushing (**b**), flexural (**c**), connector failure (buckling) (**d**).

The pairs of geometrically similar specimens should behave very similarly in the elastic range. However, they should behave differently post-cracking as the connectors fail or do not fail based on the required horizontal shear transfer. Specimens FS50-1 and FS50-2 performed similarly within the elastic region but differed in ultimate failure as intended by the reinforcement strategy. Specimen FS50-1 reached its ultimate load gradually, and the test ended when concrete was crushed on the flexural compression wythe at the load application point (as displayed in Figure 10). FS50-2 reached its ultimate capacity under approximately half the deflection reached by FS50-1, and clear connector failing sounds could be heard. 

Similar behavior was behavior was also observed for FS200-1 and FS200-2 (Figure 10a), where both specimens resulted in horizontal shear failures. Both FS200 specimens reached ultimate capacity when audible connector failures were noted, and the test was stopped. Upon post-mortem inspection, it was observed that FS200-1 experienced connector failure in the second row, whereas FS200-2 experienced connector failure at the end and second row, as shown in Figure 10a. Panel FS250-1 failed at a slightly smaller deflection but at a similar load as compared to FS250-2, and both specimens experienced connector failure, as indicated in Figure 10a. It should be noted that FS250-1 was cracked prior to testing during its removal from the formwork, so it should not be expected to behave similarly to FS250-2 in the elastic range. 

The relative slip between the two wythes was measured at the center of the four outermost connector locations. The load versus slip is plotted below for each panel in Figure 9a–f. The different slips were designated based on their location on the panel. East and west slips indicate the outermost connector locations, and east inner and west inner slips indicate the next outermost connector locations. Interestingly, the inner slips frequently were larger than the outermost slips [22,47].

FS50-1 did not experience slips as large as most of the other panels. The slip of FS50- 1 is most comparable to the slip experienced by FS200-1. Both had maximum slips of less than 12.7 mm, and the largest slips were measured on the east-inner and west-end connector locations. The slips measured for FS50-2 were larger than FS50-1, as seen in Figure 9b. The largest slips measured on panel FS50-2 occurred on the east side of the panel, with the inner east slip exceeding the slip at the furthest east connector location. The slip of FS200-1 was most comparable to the slip of FS50-1, as stated. The maximum slip experienced was less than 12.7 mm. Most of the slip occurred while the load hovered around twenty-two kN. FS200-2 experienced larger slips than panels FS50-1, FS50-2, and FS200-1 (Figure 9d). The maximum slip exceeded 40 mm, more than three times larger than the slip experienced by FS50-1 and FS200-1. The largest slips measured were recorded from the two LVDTs on the east side of the panel.

During testing, all panels developed moderate to severe flexural cracking. Most commonly, this cracking was observed symmetrically on both sides of the centerline within the section of the span experiencing the maximum moment. An example of these flexural cracks is shown in Figure 10c. Most of these cracks also occurred at or near the edge of a connector. Concrete crushing also occurred at the point of load application and is depicted in Figure 10b, but this was in all cases after horizontal shear failure. The connectors typically failed, as shown in Figure 10, similar to those in the double shear tests. FS50-1 was the only panel not to experience connector failure.

## 4. Discussion

### 4.1. Composite Action and Large-Scale Performance

The degree of composite action (DCA) is a common metric used to describe the behavior of partially composite ICSWPs in research and even for design [3]. The DCA describes how closely certain behavioral aspects of the panel perform when compared to how a panel of similar geometry would perform as non-composite or fully composite. For a non-composite panel, the concrete layers behave independently, whereas, for fully composite panels, the layers behave as a single body. DCA is frequently calculated based on the following three separate behaviors: cracking moment, ultimate moment, and deflection within the elastic region because these are historically used for the design of ICSWPs. Mathematically, this can be expressed as below.

(1) $$\mathrm{DCA}=\frac{I_{\mathrm{Exp}}-I_{\mathrm{NC}}}{I_{\mathrm{FC}}-I_{\mathrm{NC}}}$$

(2) $$\mathrm{DCA}=\frac{M_{\mathrm{Exp}}-M_{\mathrm{NC}}}{M_{\mathrm{FC}}-M_{\mathrm{NC}}}$$

where:

*I*_Exp_ = Experimental moment of inertia determined from the deflection.
*I*_NC_ = Non-composite moment of inertia determined as a sum of the moment of inertia of the individual wythes.
*I*_FC_ = Fully composite moment of inertia of the composite shape (neglects the foam contribution).
*M*_Exp_ = Experimental flexural moment.
*M*_NC_ = Non-composite flexural moment determined as the sum of moment capacities of the wythes.
*M*_FC_ = Fully composite flexural moment.

**Figure 11:**
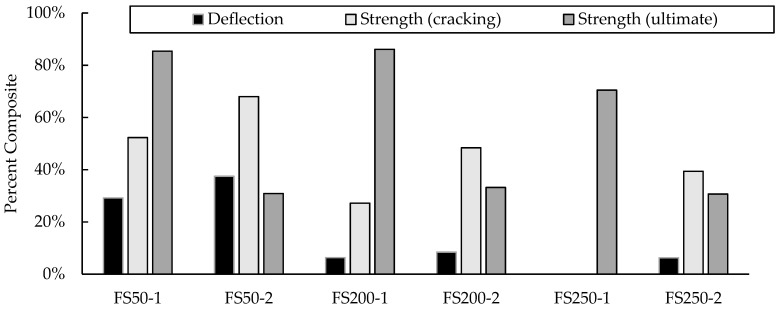
Percent composite action for cracking deflection, cracking strength, and ultimate strength.

The DCA for each panel and for each behavior described above was calculated and is shown in Figure 11. Non-composite and fully composite strengths were calculated using strain compatibility for the different cases and the measured material properties. The average measured yielding strength of the 9.5 mm rebar was 650 MPa, with a standard deviation of 80 MPa and a coefficient of variation of 12.31%, whereas the 16 mm bar had a yield strength of 457 MPa, a standard deviation of 6.75 MPa and a coefficient of variation of 1.48%. The strength and deflection based on cracking were not included for panel FS250-1 as the panel had cracked prior to testing. As can be seen in Figure 11, panels FS50-1, FS200-1, and FS250-1 all exceeded 80% composite action for ultimate strength behavior. In some cases, the excess of 100% composite action occurs in ICSWPs when comparing test values to theoretical strengths, as in the DCA calculation [46]. This phenomenon is caused by the variation of measured strengths, which are, on average, 110% for flexural members and more for shear failures [48]. 

Figure 11 shows that Series 1 panels (i.e., lightly reinforced panels) all performed at or near 80% composite action, while all Series 2 panels performed closer to 20% composite action. The difference in the composite action reached is due to the difference in steel reinforcement and the large difference in specimen depth. From Figure 11, it is shown that some panels, including panels FS200-1, FS200-2, and FS250-2, did not perform with a high percent composite action based on the deflection. These low percentages of composite action could be misinterpreted by many as an indication of ineffective partial composite action, but the low percent composite can be deceiving. Despite the low percent composite action, panels FS200-1, FS200-2, and FS250-2 were 3.6, 4.2, and 3.1 times stiffer than non-composite panels of the same dimensions. This is an artifact of dealing with the very large total depths of the 200 mm and 250 mm insulated panels, which have very high gross elastic properties. Unfortunately, using DCA to describe panel behavior can unintentionally undersell the performance of partially composite ICSWPs.

On the other hand, three elastic prediction methods were used to model the expected behavior of each of the six large-scale panels tested. The three methods used were the beam-spring method, the ISBT method, and the method developed by Holmberg and Plem [4,14,15]. The results of the large-scale testing are compared to the methods mentioned before in Table 6. These methods used material properties obtained from concrete cylinder testing, including concrete compressive strength and modulus of elasticity. The modulus of rupture of the concrete was obtained using the ACI 318-19 Table 24.5.2.1 equation for uncracked members (U) [49]. The shear stiffness of the connectors was calculated using the average of the stiffness values obtained at 0.4 times each connector’s ultimate load. Wythe thickness measurements were taken for each panel specimen. The thinner of the two wythe thickness measurements taken nearest the first observed crack was used for the thickness of the tension wythe. The depth of the wythes was taken as the depth measured at the corresponding wythe measurement used for the tension wythe. The thickness of the compression wythe was taken as the average measured wythe thickness. These dimensions were selected to model the actual behavior of the panel most accurately at cracking. There are two exceptions to the use of the above dimensions. The first exception is made for panel FS250-1, which was cracked before testing, so there was no observed first crack; instead, nominal dimensions are used for panel FS250-1 wythe thicknesses. The other exception was made because Holmberg and Plem’s method assumes equal wythe thicknesses.

The observed stiffness displayed was calculated based on the load and deflection of each panel measured at the first observed crack. The Beam Spring method and the ISBT methods agree almost identically for all panels, with the exception of panel FS250-2. All three methods adequately predict the elastic stiffness of the thick insulation panels, with the percentage difference from the observed elastic stiffness being less than 8% for all panels and methods, with only one exception. This exception is the prediction provided by Holmberg and Plem for panel FS250-2. This difference, however, can likely be attributed to some of the assumptions described earlier, namely the assumption of using the same wythe thickness for both tension and compression wythes. Panel FS250-2 dimensions were off from those specified, and the measured thickness of the tension wythe was, on average, ½ inch thicker than the compression wythe. This discrepancy between the actual panel dimensions and fitting the measured dimensions to Holmberg and Plem’s assumptions likely contributes to the overestimation of the elastic stiffness. 

Disregarding the data from FS50-2 due to the concrete section described previously, the average percent difference for each method is −3%, 1%, and −2% for the Beam Spring method, Holmberg and Plem, and ISBT method, respectively. The differences between the predicted stiffnesses are negligible because a variety of factors could result in measured results that differ within these margins of differences, such as the unintended introduction of friction. Therefore, the recommended method would be the ISBT method or the Beam Spring method. Moreover, all three elastic prediction methods can also be used to predict the cracking moment of partially composite panels. For the Beam Spring method, this is conducted by using the FEA model to find the largest stress in the beam elements in tension and using the linear relationship between load and stress to determine the load necessary for this stress to reach the modulus of rupture of the concrete. For the ISBT method, it is required to assume at what location the highest stress will accumulate and use the linear relationship between load and stress and extrapolate the applied load in the same manner as with the Beam Spring method, which will cause the stress to equal the concrete modulus of rupture. In a similar manner, Holmberg and Plem’s method can also be used to find the cracking load. This is carried out using the relationship between the force applied to the panel and the total shear force within the tension wythe. This force can be converted to stress using the cross-sectional area of the tension wythe and compared to the concrete modulus of rupture. The average percent differences between the predicted and measured cracking load are −2%, 6%, and 2% for the Beam Spring, Holmberg and Plem, and ISBT methods, respectively.

### 4.2. Horizontal Shear Strength Prediction of Large-Scale Specimens Using the Shear Flow Approach

Shear Flow is a common method used to predict the horizontal shear strength of composite members, including insulated walls. The method is based on the principles of deformable solid mechanics and compares the shear flow capacity and demand along the panel length. Common to insulated walls with discrete connectors, the shear flow capacity of a single row of connectors is calculated by dividing the connectors’ ultimate shear strength by the longitudinal connector spacing, as shown in Equation (1). The shear flow demand is calculated using the familiar mechanics of materials expression shown in Equation (2), as it is related to the applied shear loading. These equations can be found in any mechanics of materials book.

(3) $$q_{n}=\frac{F_{u}*N}{s}$$

(4) $$q_{demand}=\frac{V_{max}*Q_{FC}}{I_{FC}}$$

where:

*q*_*demand*_ = shear flow demand

*V*_*max*_ = maximum shear force due to applied load

*Q*_*FC*_ = first moment of area calculated with fully composite section properties

*I*_*FC*_ = fully composite moment of inertia

*q*_*n*_ = shear flow capacity

*F*_*u*_ = ultimate shear strength of a single connector

*N* = number of shear connectors

*s* = spacing of shear connectors

**Figure 12:**
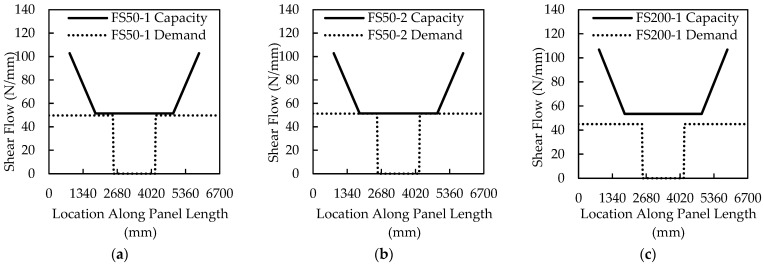

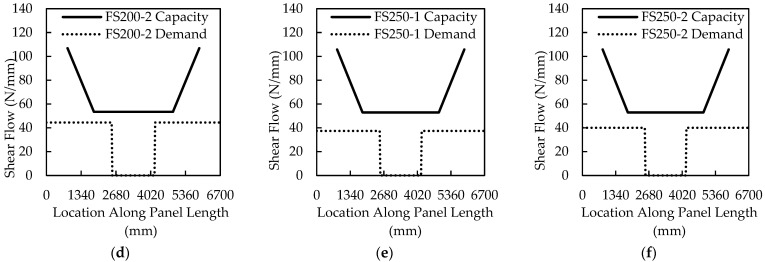
Shear flow demands and capacities along panels (**a–f**).

Figure 12 plots the shear flow capacity and demand based on the ultimate load achieved for each of the panels tested. The shear flow capacity and demand were calculated for each panel, and the results are displayed in Table 7 for the 50-mm, 200-mm, and 250-mm thick insulation panels, respectively.

The shear flow method predicted that FS50-1 and FS50-2 would fail in shear (i.e., the dashed applied shear flow and solid capacity lines in Figure 12 met or crossed). However, the shear flow method indicates that at the maximum load applied to each of the other tests, the capacity exceeded demand significantly. Shear failure was not observed in FS50- 1. The F200 and FS250 panels failed in shear but were not predicted to do so without significantly more load. In FS200-1 and FS250-1, the connector 1800 mm from the end ruptured rather than at the end, as would be expected from the shear flow model in Figure 12c,e. However, FS200-2 and FS250-2 experienced connector failure in the rows 800 mm and 1800 mm from the end, likely caused by a sudden load transfer from the loss of the 1800 mm row connector to the 800 mm row connectors.

The shear flow method did not adequately predict or describe the ultimate failure of the thick-insulation panels overpredicting the connector’s capacity for both 200-mm- and 250-mm-thick insulation panels. Table 7 presents the applied shear flow from the test, the predicted shear flow, and the measured to predicted ratio with the exception of FS50-1, which did not experience a shear failure. The measured-to-predicted ratio has an average of 0.822 and a COV of 0.11. This data indicates that the shear flow prediction may not work well for large insulation panels. There is a trend of increasing underprediction as the panel gets higher. Insulation thicknesses of 50 mm are very common, but 200 mm and 250 mm, while uncommon, are being talked about as potential options for meeting the anticipated future energy codes in the US. While outside of the scope of this paper, there seems to be a significant need for a larger investigation into the horizontal shear failure of large insulation thickness panels that the limited testing program herein cannot address.

## 5. Conclusions

Twelve double shear specimens and six large-scale panels were tested. The testing was completed to validate common engineering design methods for insulated concrete sandwich wall panels (ICSWPs) for specimens that are outside of the typical size of the industry. Specifically, specimens investigated within this research used non-proprietary connectors with panels that had thin wythes (50 mm or less) and those with thick insulation (200 mm or more). Three methods that are common in United States practice were used to model the elastic behavior of the large-scale panels and compared to the measured experimental large-scale panel testing behavior. The methods used for elastic property comparison included the ISBT method, the Beam Spring model, and a closed-form method mechanics-based method. Horizontal shear strength was predicted using shear flow and compared to the measured experimental large-scale panel testing behavior. The following conclusions can be made from the comparison of these methods and the experimental results of the large-scale panels.

- All three methods used to predict the elastic behavior of the large-scale panels were accurate in their predictions, with their average percentage differences from the measured stiffnesses within 3% for all methods.
- All three methods used to predict the cracking moment of the large-scale panels were accurate in their predictions, with their average percentage differences from the measured cracking moments within 6% for all methods.
- Based on the above bullets, the elastic methods investigated herein are suitable for the prediction of elastic deflections and cracking moments for panels with extreme dimensions in the experimental program.
- The shear flow approach did not accurately predict the ultimate capacity of the thick insulation large-scale panels, significantly overpredicting the shear capacity of the connectors with an average measured ratio of 0.82 with a COV of 0.11.

## Figures and Tables

**Table 1:** Variation of the R-value for different seasons (two extremes) as a function of the insulation thickness for a sandwich panel with 75 mm wythes and changing insulation thickness. The solid panel represents a 150 mm panel.

Insulation Thickness t_ins_ (mm)	R_winter_		R_summer_	
	(m^2^∙K/W)	(ft^2^∙°F∙h/Btu)	(m^2^∙K/W)	(ft^2^∙°F∙h/Btu)
Solid Panel	0.23	1.30	0.24	1.38
50	2.19	12.41	2.20	12.49
100	4.14	23.52	4.16	23.60
150	6.10	34.63	6.11	34.71
200	8.06	45.74	8.07	45.82
250	10.01	56.86	10.03	56.94

**Table 2:** Large-scale panel dimensions.

Panel Designation	Wythe Thicknesses (mm)	Insulation Thickness (mm)	Length (mm)	Width (mm)	Longitudinal Rebar	Transverse Rebar	Intended Failure Mode by Design
FS50-1	50	50	6700	600	2 *ϕ*9.5 mm	*ϕ*9.5 mm @350mm	*Flexure*
FS50-2	50	50	6700	600	2 *ϕ*16 mm	*ϕ*9.5 mm @350mm	*Horizontal Shear*
FS200-1	75	200	6700	600	2 *ϕ*9.5 mm	*ϕ*9.5 mm @350mm	*Flexure*
FS200-2	75	200	6700	600	2 *ϕ*16 mm	*ϕ*9.5 mm @350mm	*Horizontal Shear*
FS250-1	75	250	6700	600	2 *ϕ*9.5 mm	*ϕ*9.5 mm @350mm	*Flexural*
FS250-2	75	250	6700	600	2 *ϕ*16 mm	*ϕ*9.5 mm @350mm	*Horizontal Shear*

**Table 3:** Material testing results for double shear and large-scale specimens.

Specimen Designation	Compressive Stress (MPa)	Modulus of Elasticity (MPa)	Tensile Strength (MPa)
Double Shear Specimens			
F50-1	55.880	-	-
F50-2	55.880	-	-
F50-3	51.348	-	-
F50-4	51.348	-	-
F200-1	55.880	-	-
F200-2	55.880	-	-
F200-3	51.348	-	-
F200-4	51.348	-	-
F250-1	55.880	-	-
F250-2	55.880	-	-
F250-3	47.233	-	-
F250-4	47.233	-	-
**Large–scale Panels**			
FS50-1	65.730	35,802	3.918
FS50-2	46.809	31,944	2.894
FS200-1	65.207	35,972	4.044
FS200-2	46.809	31,944	2.894
FS250-1	63.587	38,619	3.693
FS250-2	46.809	31,944	2.894

**Table 4:** Double shear test results and their corresponding statistics.

Designation	Elastic Load (kN)	Slip at Elastic Load (mm)	Elastic Stiffness K_E0.4_ (kN/mm)	Maximum Load (kN)	Slip at Maximum Load (mm)	Ultimate Stiffness K_U0.4_ (kN/mm)
F50-1	23.60	0.65	36.57	58.98	2.50	23.59
F50-2	21.14	0.72	29.25	52.84	2.75	19.22
F50-3	20.04	0.59	33.95	50.09	2.50	20.03
F50-4	18.79	0.52	36.01	46.97	2.00	23.49
**Mean**	**20.89**	**0.62**	**33.95**	**52.58**	**2.44**	**21.58**
**COV (%)**	**8%**	**12%**	**8%**	**8%**	**11%**	**9%**
F200-1	21.22	0.89 **	23.83 **	53.07	9.65 **	5.50 **
F200-2	21.04	1.52	13.81	52.58	6.35	8.28
F200-3	25.13	2.03	12.37	62.85	6.35	9.90
F200-4	18.95	1.78	10.66	47.33	6.10	7.76
**Mean**	**21.71**	**1.78**	**12.28**	**54.25**	**6.27**	**8.65**
**COV (%)**	**10%**	**12%**	**10%**	**10%**	**2%**	**11%**
F250-1	21.92	2.83	7.75	55.19	9.25	5.97
F250-2	22.03	1.90	11.63	55.46	6.50	8.53
F250-3	19.02	2.24	8.50	47.89	7.25	6.61
F250-4	22.99	2.56	8.98	57.88	7.75	7.47
**Mean**	**21.49**	**2.38**	**9.22**	**54.11**	**7.69**	**7.14**
**COV (%)**	**7%**	**15%**	**16%**	**7%**	**13%**	**13%**

** Value not included in mean and COV calculation because deflection sensor was deemed anomalous.

**Table 5:** Failure types of tested large-scale panels.

Panel Designation	Applied Load at Failure	Applied Shear at Failure	Applied Moment at Failure	Failure Mode
	kN	kN	kN·m	
FS50-1	10.7	5.34	11.4	flexural
FS50-2	10.9	5.47	11.7	shear
FS200-1	25.6	12.8	27.3	shear
FS200-2	25.3	12.6	26.9	shear
FS250-1	25.0	12.5	26.7	shear
FS250-2*	25.1	12.6	26.8	shear
* Values for F250-2 are for the post-cracking peak.				

**Table 6:** Comparison of large-scale panel behavior to analytical methods.

Panel Designation	Experiments		Beam Spring Method		Holmberg and Plem		ISBT Method	
	Observed Stiffness (kN/mm)	Measured M_c_(kN-m)	Predicted ÷ Observed Stiffness	Predicted ÷ Observed Mcr	Predicted ÷ Observed Stiffness	Predicted ÷ Observed Mcr	Predicted ÷ Observed Stiffness	Predicted ÷ Observed Mcr
FS50-1	0.51	4.98	0.83	1.10	0.83	1.14	0.83	1.17
FS50-2	0.54	4.63	0.71	0.99	0.74	1.10	0.71	1.00
FS200-1	1.35	12.22	1.06	1.10	1.06	1.18	1.05	1.15
FS200-2	1.49	15.03	0.93	0.79	0.98	0.95	0.94	0.84
FS250-2	1.52	14.86	1.03	0.90	1.18	0.99	1.08	0.94

**Table 7:** Standard and modified shear flow measured to predicted comparisons.

Panel Designation	Standard Shear Flow Procedure	
	q_u_(kN/mm)	Measured/Predicted
FS50-1	49.62	-
FS50-2	50.98	0.99
FS200-1	45.40	0.85
FS200-2	44.54	0.83
FS250-1	37.35	0.71
FS250-2	38.64	0.73

## Data Availability

Data will be made available upon reasonable request.
